# Balancing public health needs and economic sustainability: A dual-matrix model for community pharmacy inventory management

**DOI:** 10.1016/j.rcsop.2026.100727

**Published:** 2026-03-03

**Authors:** Ana Golić Jelić, Valentina Topić Vučenović, Saša Vučenović, Vanda Marković-Peković, Amanj Kurdi, Brian Godman, Johanna C. Meyer, Ranko Škrbić

**Affiliations:** a Faculty of Medicine, Department of Pharmacy, University of Banja Luka, 78000 Banja Luka, Bosnia and Herzegovina; b Faculty of Economics, University of Banja Luka, 78000 Banja Luka, Bosnia and Herzegovina; c Department of Public Health Pharmacy and Management, School of Pharmacy, Sefako Makgatho Health Sciences University, Molotlegi Street, Garankuwa, Pretoria 0208, South Africa; d College of Pharmacy, Hawler Medical University, Erbil, Kurdistan Region, Iraq; e College of Pharmacy, Al-Kitab University, Kirkuk 36015, Iraq; f South African Vaccination and Immunisation Centre, Sefako Makgatho Health Sciences University, Molotlegi Street, Garankuwa, Pretoria 0208, South Africa; g Faculty of Medicine, University of Banja Luka, 78000 Banja Luka, Bosnia and Herzegovina; h Academy of Sciences and Arts of the Republic of Srpska, 78000 Banja Luka, Bosnia and Herzegovina

**Keywords:** Community pharmacy, Inventory management, ABC, FSN, Profitability, Gross profit, Republic of Srpska

## Abstract

**Background:**

Community pharmacies must balance public health obligations with economic sustainability. However, integrated methods that jointly manage medical and non-medical inventory in community pharmacies in LMICs are limited.

**Objective:**

To develop and apply a dual-matrix model separating medical from non-medical products into operational control categories and introducing a High–Medium–Low profitability (HML-P) classification.

**Methods:**

We conducted a retrospective, descriptive analysis of all items handled in six community pharmacies in the Republic of Srpska, Bosnia and Herzegovina, during the analyzed 2022 year (12-month period) (n = 10,541). Medical products were classified by Always Better Control (ABC) by purchase value and Fast-/Slow-/Non-moving (FSN) by dispensing frequency (predefined thresholds: >4/day = F, 1–4 = S, <1 = N) to form an ABC–FSN matrix. Non-medical products were classified by ABC and a new HML-P scheme (expert-defined Pareto cut-offs: 70%/20%/10% of cumulative gross profit) to form an ABC–HML-P matrix. Each matrix was consolidated into three control categories: I (strict), II (moderate) and III (minimal).

**Results:**

Non-medical products constituted 76.4% of all items. The ABC–FSN matrix identified Im = 149 medical products for strict control, while the non-medical ABC–HML-P matrix identified Inm = 580 items for strict control and a large segment for minimal oversight (IIInm = 6218). A pronounced Pareto pattern was observed (≈10% of items accounted for 70% of spend and 70% of gross profit), alongside low daily movement (only 3.2% dispensed ≥1/day).

**Conclusions:**

The proposed dual-matrix model provides a practical decision-support tool for community pharmacies. It helps prioritize availability of patient-critical medical products while supporting economic sustainability.

## Highlights

• Introduction of a dual-matrix model for community pharmacy inventories.
• Separation of medical (ABC–FSN) from non-medical (ABC–HML-P) products.
• Mapping items to control Categories I–III for focused stock oversight.
• Framework for balancing public-health priorities with economic sustainability in LMICs.

## 1 Introduction

Community pharmacies, as integral healthcare institutions, play a crucial role in public health by ensuring the supply and dispensing of medical products. They are also mandated to participate in health promotion, disease prevention, and patient education.^1^ Moreover, they are the first healthcare professional (HCP) contact for self-limiting infectious conditions such as upper respiratory tract infections (URTIs), including during the COVID-19 pandemic, which is particularly important in low- and middle-income countries (LMICs).^2^, ^3^, ^4^, ^5^, ^6^, ^7^, ^8^, ^9^ While non-prescription antibiotic dispensing and patient pressure remain concerns in a number of LMIC settings, stewardship activities in the Republic of Srpska have contributed to reduced antibiotic dispensing for URTIs and relatively stable antibiotic use.^8^, ^9^, ^10^, ^11^, ^12^, ^13^, ^14^, ^15^, ^16^, ^17^ In view of ongoing concerns with access to HCPs in public primary healthcare clinics, we are also seeing community pharmacists in a number of countries allowed to treat and dispense an agreed list of antibiotics.^8^, ^18^, ^19^, ^20^, ^21^, 22. In parallel, community pharmacists contribute to non-communicable disease (NCDs) care through patient counselling (e.g., dosing schedules, inhaler technique and adherence support), as well as advice on pain management and suitable alternatives during shortages.^18^, ^19^, ^20^, ^21^, 22., 23, 24, 25, 26, 27, 28, 29, 30, 31

At the same time, pharmacies must function as business entities for their survival, alongside advising and dispensing medical products, including managing a broad assortment of non-medical items. This dual role requires rational and goal-oriented decision-making to enhance operational efficiency and ensure a sustainable performance, while at the same time aligning public health responsibilities with long-term economic viability. Given these dual obligations and the heterogeneous assortment, we conceptualize community pharmacy inventory as two distinct sub-systems—medical products that must be managed primarily by public-health needs and non-medical products that must be managed primarily by economic contribution—requiring differentiated inventory-control approaches.

In Bosnia and Herzegovina, pharmacy practice is regulated at the constituent entity level (The Republic of Srpska and Federation of Bosnia and Herzegovina). In the Republic of Srpska, the Pharmacy Practice Law and related regulations govern the scope of pharmacy services, authorizing the dispensing of medicines, medical devices, baby food and equipment, dietary supplements, cosmetics, and other health-related products.32., 33 The Agency for Medicinal Products and Medical Devices of B&H (ALIMBiH) establish maximum wholesale prices for prescription medicines across both entities,^34^ while the Government of the Republic of Srpska additionally regulates maximum margins for all medicines. Consequently, community pharmacies cannot simply adjust prices or mark-ups to improve the profitability of dispensed medicines. Community pharmacies must also operate in compliance with this legal framework and the principles of Good Pharmacy Practice to ensure that decision-making reconciles economic viability with ethical responsibilities.32., 35., 36., 37. In the Republic of Srpska, pharmacies are also legally authorized to provide a broad array of health-related products. These include medical devices, baby food, supplements, cosmetics and over the counter (OTC) medicines. As a result, community pharmacies in the Republic must optimize their operations and product mix to remain financially viable. Similar situations and challenges exist in other countries leading for instance to appreciable closures of community pharmacies in the UK in recent years, following significant real-terms cuts in funding for the dispensing of medicines. This has resulted in urgent discussions to address the situation with a commitment for increased funding.38., 39.

In view of this, inventory management in community pharmacies plays a critical role in both operational efficiency and the quality of patient service delivery. Effective pharmaceutical inventory management encompasses processes ranging from procurement and storage to dispensation and continuous monitoring of stock levels.^40^ These activities are essential not only to guarantee uninterrupted access to medical products for patients but also to optimize the allocation of financial resources for continued viability.^41^, ^42^ However, pharmacies today encounter numerous challenges in managing inventories efficiently.^40^ These include fluctuating demand patterns, evolving regulatory requirements, frequent supply chain disruptions especially among LMICs, and the imperative to control costs whilst maintaining adequate stock levels.^40^, ^43^, ^44^, ^45^, ^46^, ^47^ Poor inventory management practices may lead to stockouts, overstocking, increased operational costs, higher levels of pharmaceutical waste, and ultimately, compromising patient care.^40^, ^48^, ^49^, ^50^

Established classification systems and matrix-based approaches have been documented to help with inventory management particularly for hospital settings, where such methods provide structured methodologies for analyzing, prioritizing, and optimizing pharmaceutical stock.^49^, ^51^, ^52^, ^53^, ^54^, ^55^ Many employ classification methods including the Always, Better, Control (ABC), the Vital, Essential, and Desirable/Not essential (VED/VEN), the Fast-moving/Slow-moving/Non-moving (FSN), and similar techniques, as well as their matrices that support evidence-informed decision-making.^49^, ^51^, ^52^, ^55^, ^56^, ^57^ Such approaches have demonstrated their effectiveness in hospital settings,^58^ with improved systems also being introduced across sectors in LMICs to improve stock control generally.^59^

However, evidence in community pharmacies where inventories include both medical and non-medical items and where pharmacies must simultaneously meet public health obligations and maintain economic viability, remains limited.^60^, ^61^ In particular, integrated inventory models that explicitly combine these two objectives and translate them into actionable control categories for heterogeneous assortments are still lacking, especially in LMICs. Inventory management in this setting requires managing the wide range of products with careful planning, design, and organizational strategies to ensure sustainable functioning in the face of continual financial pressures, while addressing the needs of a high volumes of patients daily. These factors underscore the complexity of community pharmacies and the necessity of developing tailored approaches that reconcile public health objectives with economic sustainability, alongside enabling pharmacy professionals to devote sufficient time and attention to patient care. This means improving on the current models^62^ to include both categories of available products and services. Medical products need to be viewed from the perspective of their public health significance and non-medical products from the perspective of convenience to patients and profitability. Operationally, this distinction can be translated into selective inventory control: medicines of high public health significance warrant stronger availability safeguards and more frequent review, whereas non-medical items can be prioritized by movement and contribution to support financial sustainability under limited working capital and regulated pricing. The dual-matrix approach used in this study provides a simple, implementable way to map these priorities into actionable control tiers for routine pharmacy inventory management.

Consequently, the objectives of this study were to (i) retrospectively characterize inventory patterns in a healthcare facility comprising six community pharmacies in the Republic of Srpska, (ii) establish a practical distinction between medical and non-medical product categories within community pharmacy inventories, and (iii) refine existing inventory management frameworks by developing and applying a dual-matrix approach, consolidated into operational control categories**,** through tailored and easy-to-implement management strategies that balance public health priorities with economic sustainability**,** with potential applicability beyond the Republic of Srpska to similar contexts.

## 2 Methodology

### 2.1 Study design and data collection

This retrospective study was based on routinely collected data obtained from a healthcare facility comprising six community pharmacies that operate under a single administrative organization in the Republic of Srpska. The pharmacies served both urban and rural catchment areas and included locations in different service environments. The facility handled over 10,000 different products during the analyzed year 2022 (1 January–31 December 2022).

The final database for the study included the following for each stocked item: product code, product name, quantity purchased, quantity dispensed, purchased value excluding Value Added Tax (VAT), margins per product (adjusted for discounts and promotions), pharmaceutical service (dispensing of prescription medicines and reconstitution of medicines) fee reimbursed by the Public Health Insurance Fund of Republic of Srpska (PHIF), manufacturer's name, and type of product. The database did not include a data column capturing essential medicines list status, as this functionality was not supported by the facility's software.

#### 2.1.1 Medical vs. Non-medical product categorization

For the purpose of this study, two main categories of products were defined: (1) the medical products category, i.e. all items registered by ALIMBiH (prescription and OTC medicines and medical devices),^34^ and (2) non-medical products category which comprised all remaining items sold or dispensed within the six community pharmacies.

The healthcare facility employed an internal inventory classification tailored to operational needs. Within this scheme, the medical products category included prescription medicines, OTC medicines, and medical devices. Non-medical products category included facility's internal classes: non-medical devices, teas, cosmetics, dermocosmetics, baby food, baby equipment, dietary supplements, homeopathic products, glasses, footwear, bandaging materials and food for special patient populations, e.g. those with diabetes mellitus or gluten intolerance.

## 3 Data analysis

### 3.1 Inventory grouping into classes

#### 3.1.1 ABC classification based on total purchased value

All products were categorized into classes using the ABC method,^63^ based on Pareto's principle that a relatively small number of items account for the majority of expenditure. According to this approach, products that consume 70% of the total budget are classified as Class A, those consuming 20% as Class B, and the remaining 10% as Class C.^61^, ^63^

#### 3.1.2 FSN classification based on dispense frequency patterns

Products were also classified using the FSN method, which groups items according to their dispensing frequency. Class F includes fast-moving products dispensed most frequently, Class S covers slow-moving products with moderate dispensing, while Class N comprises non-moving products, often referred to as dead stock.^41^, ^64^ In community-pharmacy assortments, low or intermittent daily movement is common across many items, and FSN classification is used here primarily to support prioritization of routine monitoring within the observed assortment. In this study, the specific thresholds were derived a priori from the literature^64^ and subsequently verified through consensus by an expert panel of five health facility pharmacists with more than five years of community pharmacy experience, reflecting local workflows and dispensing patterns. Products dispensed more than four times per day were categorized as Class F; products dispensed one to four times per day as Class S; and products dispensed less than once per day as Class N.

#### 3.1.3 Creation of new high/medium/low profitability classification method for non-medical products based on gross profit

A new classification framework was developed from an economic perspective, namely High/Medium/Low profitability (HML—P) classification. This classification was created for the purpose of this study in order to categorize pharmacy non-medical inventories according to their product profitability given the aims and objectives of the study. This was measured by their gross profit. Gross profit represents the gross revenue retained by the pharmacy from the sale of a product, calculated as the difference between its retail price and purchase price (PHIF-reimbursed service fees and any discounts/promotions were incorporated; VAT did not affect the calculated difference). This difference reflects the financial outcome achieved by the pharmacy after covering the acquisition cost of the product and serves as a key indicator of economic sustainability and profitability. Within this system, high profitability (H) products represent items with the greatest cumulative profit and the most substantial contribution to the financial sustainability of the pharmacy over a year, i.e. accounting for 70% of total gross profit. Medium profitability (M) products (20% of total gross profit) constitute items with moderate profitability, typically forming the backbone of the assortment of available products in the pharmacy with stable turnovers. Low profitability (L) products (10% of total gross profit) encompass low-margin items that provide limited direct economic return. However, they may be necessary for fulfilling patients' needs or maintaining a comprehensive product offering. We derived these thresholds from ABC classification and its principles.

**Fig. 1:**
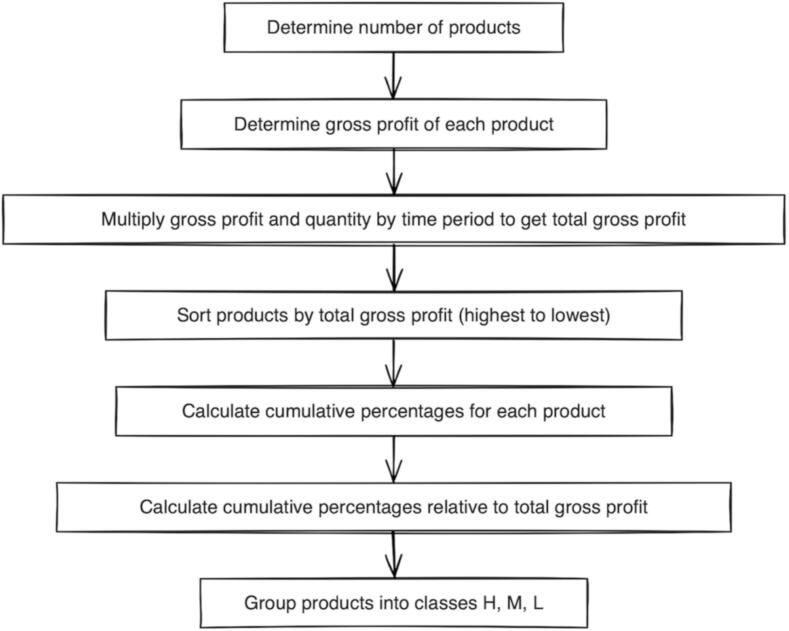
Steps of HML-P inventory classification based on gross profit.

An inventory grouping process according to the HML-P classification was performed in a number of steps. These are presented in Fig. 1.

### 3.2 Inventory grouping into matrices

Two matrices were constructed for the purposes of this study. The first matrix combined the ABC and FSN classifications and was applied exclusively to medical products. In this matrix items were prioritized based on patient needs (FSN classification) and their contribution to the budget expenditure (ABC classification). The second matrix applied only to non-medical products combining the ABC and HML-P classifications. It highlights different levels of monitoring requirements based on profitability (HML-P classification) and budget impact (ABC classification).

Based on the intersection of the two classification methods, all the products in the inventory were initially allocated into subgroups corresponding to the individual cells of the resulting matrices. Each subgroup represented a distinct combination of consumption value criteria (ABC) vs. dispensing frequency (FSN)/ profitability (HML—P) criteria for medical/non-medical products.

For the purposes of inventory control^61^ matrix cells were consolidated into Categories I–III using selective control logic, whereby Category I includes cells belonging to the highest-priority class on either axis (e.g., high expenditure or high movement/profitability), while Categories II and III reflect progressively lower combined priority:

• Category I (Strict control): subgroups containing items of budget-intensive nature and high public health importance/substantial contribution to profitability (products in cells: AF/H, AS/M, AN/L, BF/H, and CF/H). These products should receive close monitoring and tight inventory control to ensure efficient use of resources and continuous availability.
• Category II (Moderate control): subgroups consisting of items with moderate importance, which typically warrant balanced monitoring without intensive control efforts (BS/M, BN/L, and CS/M cells).
• Category III (Minimal control): subgroup (cell CN/L) characterized by low importance, low profitability, or lack of consumption (dead stock). These products can typically be managed with minimal monitoring and may be considered for substitution, reduction, or elimination.

These categories are intended as empirical decision-support tiers indicating the recommended intensity of inventory oversight. They are not presented as mandatory rules, and pharmacies may adapt thresholds and actions based on local demand shifts, procurement constraints, and legal/clinical obligations. Category assignment is derived descriptively from observed purchasing/dispensing (medical items) and gross-profit patterns (non-medical items), while the suggested “control intensity” is normative guidance aimed at practical implementation.

This approach enabled the structuring of medical products into control Categories Im, IIm, and IIIm, and non-medical products into control Categories Inm, IInm, and IIInm (Table 1).

**Table 1:** Mapping of matrix subgroups to inventory control categories (Category I–III).

Class	F/H	S/M	N/L
A	I_m/nm_	I_m/nm_	I_m/nm_
B	I_m/nm_	II_m/nm_	II_m/nm_
C	I_m/nm_	II_m/nm_	III_m/nm_

m = medical; nm = non-medical.

Im/nm = strict control; IIm/nm = moderate control; IIIm/nm = minimal control.

A/B/C = purchase-value class.

F/S/N = dispensing-frequency class.

H/M/L = profitability class.

### 3.3 Inventory turnover ratio and days inventory held

The inventory turnover ratio reflects the number of times, on average, inventory is replenished during a year. In this study, the turnover ratio was calculated for the overall inventory as well as separately for each ABC class, using the following formulas:

Inventory Turnover Ratio=Total annual purchased value/Average inventory balance

$$\text{Average inventory balance}=\left(\text{Inventory}\;\mathrm{at}\;\text{the beginning of the year}+\text{Inventory}\;\mathrm{at}\;\text{the}\;\mathrm{end}\;\text{of the year}\right)/2$$

Days inventory held, representing the average duration of one turnover cycle, was calculated using the formula: 365 / Inventory Turnover Ratio.

## 4 Results

Results are presented in four steps: (i) overall inventory composition, (ii) single-dimension classifications (ABC, FSN, and HML—P), (iii) matrix-based control categories for medical and non-medical products (ABC–FSN and ABC–HML-P), and (iv) inventory turnover metrics (ratio and days inventory held).

### 4.1 Overall inventory composition

All the medical and non-medical products (*N* = 10,541) were analyzed of which 2490 (23.6%) were medical and 8051 (76.4%) were non-medical products.

### 4.2 Single-dimension classifications

The ABC classification was performed based on the purchase value of 10,541 medical and non-medical products into three classes, with Class A accounting for the majority of total purchased value, whereas the largest share of items falls into Class C with a comparatively small value share (Table 2).

**Table 2:** ABC Analysis of Medical and Non-Medical Products.

Class	Total Purchased Value per Product (EUR)	Number of Products	% of Total Number of Products	Cumulative Purchased Value (EUR)	% of Total Purchased Value
A	> 573	1102	10.5	2,016,263	70.0
B	153–573	1887	17.9	576,075	20.0
C	< 153	7552	71.6	288,037	10.0

The FSN analysis showed that 339 products (3.2%) were dispensed at least once per day (classes F and S), accounting for 29.0% of the total purchase value of all products. The vast majority of medicines held in the pharmacies were dispensed less than once per day (Table 3).

**Table 3:** FSN Analysis of Medical and Non-Medical Products.

Class	Dispense Rate (number of products dispensed per day)	Number of Products	% of Total Number of Products	Purchased Value (EUR)	% of Total Purchased Value
F	>4	64	0.6	188,858	6.6
S	1–4	275	2.6	622,651	21.6
N	< 1	10,201	96.8	2,068,866	71.8

As a result of the HML-P analysis only 9.6% of products accounted for 70% of the total value achieved in the healthcare facility, whereas 73.0% of products contributed to 10% of the total gross profit (Table 4).

**Table 4:** HML-P Analysis of Medical and Non-Medical Products.

Class	Number of Products	% of Total Number of Products	Total gross profit in EUR	% of Total gross profit
H	1009	9.6	484,938	70.0
M	1830	17.4	138,643	20.0
L	7701	73.0	69,326	10.0

### 4.3 Matrix-based control categories for medical and non-medical products

#### 4.3.1 ABC-FSN matrix analysis for medical products

Table 5 presents the allocation of medical products into subgroups corresponding to the individual cells of the ABC–FSN matrix. It also illustrates the consolidation of these subgroups into three control categories (Im, IIm, and IIIm), reflecting different levels of monitoring requirements. Categories Im (strict control), IIm (moderate control) and IIIm (minimal control) included 149, 207 and 2134 medical products respectively.

**Table 5:** ABC-FSN Matrix for Medical Products.

Class	F	S	N
A	22	74	39
B	3	46	126
C	11	35	2134

A/B/C = purchase-value class.

F/S/N = dispensing-frequency class.

AF, BF, CF, AS, AN = Category Im (strict control).

BS, BN, CS = Category IIm (moderate control).

CN = Category IIIm (minimal control).

*ABC-HML-P Matrix Analysis for Non-Medical Products.*

ABC-HML-P matrix resulted in 580, 1251 and 6218 non-medical products in categories Inm, IInm and IIInm respectively (Table 6).

**Table 6:** ABC and HML-P Matrix for Non-Medical Products.

Class	H	M	L
A	201	0	1
B	247	37	3
C	131	1211	6218

A/B/C = purchase-value class.

H/M/L-P = profitability class.

AH, BH, CH, AM, AL = Category Im (strict control).

BM, BL, CM = Category IIm (moderate control).

CL = Category IIIm (minimal control).

### 4.4 Inventory turnover metrics

The overall inventory turnover ratio was 4.6, while the average number of days inventory was held was 78.6 days. This indicates a moderate holding period for a mixed community-pharmacy assortment. A more detailed analysis by ABC classification reveals that the products making up the largest portion of the pharmacists' budget (Class A) also had the highest turnover ratio, resulting in the lowest number of days inventory held (Table 7). On the other hand, Class C products have the lowest turnover ratio, with their average holding time being as long as 134 days.

**Table 7:** The average inventory turnover ratio by ABC classification.

Class	Number of products	% of Total Number of Products	% Total Purchased Value	Turnover ratio	Average number of days inventory held
A	1102	10.0	70.0	11.2	32.5
B	1887	18.0	20.0	9.3	39.3
C	7552	72.0	10.0	2.7	134.7

## 5 Discussion

This study proposes a new framework to inventory management in community pharmacies among LMICs that combines existing ABC and FSN classifications with a new HML-P classification based on gross profit as well as distinguishing medical from non-medical product lines.

The central development is the instigation of the HML-P classification derived from the gross profit of items held within the surveyed community pharmacies. In contrast to traditional ABC analyses, the HML-P approach evaluates profitability, which is particularly useful, for community pharmacies operating under regulated margins and financial constraints. When integrated into the ABC–HML-P matrix for non-medical products, this profitability perspective appears to become particularly salient, directing managerial attention to the items that most sustain the institution's financial health. As a result, this combined approach could provide practical value in optimizing inventory management. This is particularly relevant in LMICs, where community pharmacies are often a key component of primary care, including being a first point of contact for self-limiting conditions such as URTIs; appropriate advice can reduce unnecessary antibiotic use and help mitigate antimicrobial resistance.^9^, ^10^, ^21^ Community pharmacies also support patients with chronic NCDs through adherence support, counselling on correct medicine use (e.g., inhaler technique), and lifestyle advice, which is especially important where access to other healthcare providers may be limited. Strengthening the capacity of community pharmacies in these dual areas is increasingly essential, not only for improving patient outcomes in LMICs, where access to other healthcare providers may be limited, but also for reinforcing the resilience and sustainability of the broader healthcare system.

In our study only 10.5% of products account for 70% of the healthcare facility's total budget aligning with Gupta et al.^56^ and Mfizi et al^57^ These high-value items were being managed relatively efficiently with Class A showing an annual turnover rate of 11.2, i.e. approximately 32 days of stock on hand. This is appreciably higher than the overall average annual turnover of 4.6, indicating that the surveyed pharmacies were already practicing good stock control, i.e. frequent, smaller purchases in line with internal recommendations for their top budget items to avoid tying up excessive capital. In contrast, Class C items, i.e. low-expenditure items accounting for 72.0% of the total number of items, had a relatively sluggish turnover at approximately 2.7, or 134 days of stock on hand. This suggests that most of the products available in the surveyed pharmacies were overstocked relative to demand, locking in resources and incurring the risk of expiry before being dispensed or sold.

The FSN analysis revealed that only 339 products (3.2%) belong to categories F and S (dispensed at least once daily), accounting for 29.0% of total purchase value. This means that approximately 97% of items in the pharmacies were “Non-moving” on a daily basis, consuming over two-thirds of the budget. This imbalance implies considerable inefficiency in inventory investment that also needs to be addressed going forward to improve the viability of community pharmacies. The low percentage of fast and slow-moving products compared to non-moving products reflects the unique challenges faced by community pharmacies in maintaining comprehensive product availability while managing financial resources efficiently.^41^ This pattern differs significantly from typical retail environments and underscores the need for specialized inventory management approaches in community pharmacy settings.

Our categorization of medical and non-medical products recognizes that medical products should be managed with a priority of public health significance since pharmacies must stock even low-margin essential drugs as part of their mandate, whereas non-medical products can be leveraged to improve earnings within the allowed framework. Furthermore, defining control categories from the matrices provided a structured and pragmatic framework for operational decision-making, allowing complex outputs from both matrices to be translated into actionable levels of inventory control, tailored to the specific characteristics of medical and non-medical products in community pharmacy settings. Results of the ABC-FSN matrix analysis align with the study by Gupta et al.^56^, who used an ABC-VED matrix and achieved similar management categories. ABC–VED/VEN approaches^56^, ^57^ are useful for prioritizing items by value combined with criticality; however, they do not explicitly separate medical and non-medical assortments or incorporate a profitability dimension for non-medical products. Our dual-matrix model was designed to remain implementable using routinely available product data in community pharmacies, while translating outputs into operational control categories tailored to the distinct roles of medical and non-medical product lines.

From the aspect of continuity of healthcare service provision, it is especially important that Category Im products, including those from AF, AS, AN, BF, and CF matrix cells, i.e. those products with high budget impact (A category) and those with high dispensing frequency (F category) are prioritized. This approach ensures that important products that are frequently dispensed are not neglected simply because they have low individual value. However, for the financial sustainability of community pharmacies, we believe it is particularly important to integrate a profitability-based categorization (HML-P analysis) alongside a traditional consumption/value metrics (ABC) for non-medical products. Our ABC-HML-P matrix showed that only 7.2% of non-medical products demand strict control, 15.5% call for moderate control, while the vast majority (6218 products) need only minimal oversight. This categorization enables pharmacy owners to principally focus on non-medical products that contribute most to their profitability, which is crucial for economic sustainability of community pharmacies. By identifying Category Inm items that combine high margins and high revenue contribution, community pharmacies in LMICs can prioritize these for availability and promotional focus to maximize their income. Simultaneously, low-profit items (Category IIInm) that contribute little financially can be minimized or replaced with more profitable alternatives if they are not critical to patient care. To support day-to-day implementation of these control tiers, pharmacies may link each control category to a small set of routine actions. For example, Category I items (Im/Inm) could be reviewed weekly (or at each ordering cycle) with minimum-stock safeguards and stock-out alerts to ensure continuous availability. Category II items (IIm/IInm) could be managed with monthly review and standard replenishment rules. Category III items (IIIm/IIInm) may be checked quarterly, with non-moving items triggering a delisting/substitution review and replenishment only when justified by clinical/legal obligations or specific demand.

Overall, the proposed framework of ABC/FSN for medical and ABC/HML-P for non-medical products appears to balance the ethics and economics of community pharmacies through helping to ensure that medical products are managed in accordance with their crucial healthcare role, while non-medical products are managed for profit optimization to subsidize the pharmacy's public health mission. In practice, pharmacy managers must balance competing objectives: safeguarding availability and maintaining a broad assortment, particularly for patient-critical medicines, while limiting capital tied-up in inventory, holding costs, and expiry risk. The proposed control tiers are intended to support transparent prioritization across these trade-offs within the local regulatory and budget context. This balanced strategy is particularly relevant in settings where pharmacies operate with limited margins for prescribed medicines and expanding public health roles. By reallocating inventory investment toward high-yield items, and reducing capital tied-up in under-performing stock, a community pharmacy can improve its financial health without compromising patient access to required therapies. Moreover, a more efficient inventory management directly translates into cost savings and improved income, strengthening the pharmacy's ability to remain solvent and invest in quality improvement at a time of continuing pressures. Strengthening control of Category I medical items may reduce stock-outs and help improve equity of access, particularly for vulnerable patient groups, while in implementation, the profitability lens for non-medical items can be used in a way that supports financial sustainability without detracting from patient-critical priorities.

We are aware though of several limitations with this study. First, we did not incorporate a VED classification for medicines since the pharmacy's software did not indicate essential medicines needed for that analysis. Clinical importance was also not explicitly factored into our matrices. Secondly, our analysis was retrospective and descriptive in nature. However, we have identified inefficiencies and proposed an optimized categorization to help going forward. Consequently, while we draw on logical inference and comparisons to suggest improvements, we did not perform a before–after implementation evaluation, thus the actual impact of adopting this model in practice remains to be empirically validated. Moreover, the dataset did not include patient-level or service-quality outcomes, so we could not assess whether the proposed approach translates into measurable improvements in care. Finally, the proposed framework is primarily intended for community pharmacy inventory structures, and its applicability to other settings (e.g., hospital pharmacies or non-pharmacy retail) may be more limited. Future research should therefore include pilot implementation studies, longitudinal evaluation of performance over time, and comparisons against standard inventory practices to assess real-world impact. This dual-matrix framework could also be expanded by adding a VED dimension similar to the situation in hospitals or by updating the model periodically to capture shifting demand trends.

From a policy perspective, adoption of such a model across community pharmacies could standardize and greatly improve inventory practices, contributing to overall health system efficiency among LMICs. In practical terms, implementation could be supported through integration into existing procurement/stock-management software or through software with minimal requirements (e.g., an Excel-based template), brief staff training on Category I–III actions, and monitoring using simple indicators (e.g., stock-outs in Category Im items and gross-profit contribution of Category Inm items).

Despite the limitations, we believe this classification model is easy to implement and offers immediate value for decision-makers and pharmacists. The proposed model employs standard inventory data and well-established analysis techniques, making it relatively easy to adopt without sophisticated technology. The output is straightforward to interpret and action, allowing pharmacy managers to integrate these categories into their procurement and stock review cycles if wished.

## 6 Conclusion

In conclusion, this study proposes a dual-matrix decision-support framework for inventory management in community pharmacies as a practical tool for bridging the gap between pharmaceutical care obligations and business sustainability given the current financial pressures on community pharmacies across countries. By implementing separate control matrices for medical and non-medical products, our proposed framework translates these classifications into operational control actions that can support structured inventory oversight under regulated margins and financial constraints. While the study is retrospective and descriptive, the approach offers a practical basis for standardizing inventory control across medical and non-medical assortments in community pharmacy settings especially in LMICs.

During the preparation of this work the authors used ChatGPT in order to improve the writing process. No AI tools were used for data analysis or decision-making. After using this tool/service, the authors reviewed and edited the content as needed and take full responsibility for the content of the published article.

## CRediT authorship contribution statement

**Ana Golić Jelić:** Writing – review & editing, Writing – original draft, Visualization, Supervision, Methodology, Investigation, Formal analysis, Data curation, Conceptualization. **Valentina Topić Vučenović:** Writing – review & editing, Writing – original draft, Visualization, Supervision, Methodology. **Saša Vučenović:** Writing – original draft, Methodology, Formal analysis, Data curation. **Vanda Marković-Peković:** Writing – review & editing, Writing – original draft, Methodology. **Amanj Kurdi:** Writing – review & editing, Formal analysis. **Brian Godman:** Writing – review & editing, Methodology, Formal analysis. **Johanna C. Meyer:** Writing – review & editing. **Ranko Škrbić:** Writing – review & editing, Methodology, Conceptualization.

## Declaration of competing interest

The authors declare that they have no known competing financial interests or personal relationships that could have appeared to influence the work reported in this paper.
